# Prevalence of Meeting Aerobic, Muscle-Strengthening, and Combined Physical Activity Guidelines During Leisure Time Among Adults, by Rural-Urban Classification and Region — United States, 2020

**DOI:** 10.15585/mmwr.mm7204a1

**Published:** 2023-01-27

**Authors:** Christiaan G. Abildso, Shay M. Daily, M. Renée Umstattd Meyer, Cynthia K. Perry, Amy Eyler

**Affiliations:** ^1^West Virginia University School of Public Health, Morgantown, West Virginia; ^2^West Virginia University Office of Health Affairs, Morgantown, West Virginia; ^3^Institute for Behavioral Health, Brandeis University Heller School for Social Policy and Management, Waltham, Massachusetts; ^4^Baylor University, Robbins College of Health and Human Sciences, Waco, Texas; ^5^Oregon Health & Science University, School of Nursing, Portland, Oregon; ^6^Prevention Research Center in St. Louis, Brown School at Washington University in St. Louis, St. Louis, Missouri.

The healthful effects of physical activity on a multitude of physical and mental health outcomes are well documented (*1*). Despite promising increases in the percentage of U.S. adults meeting aerobic and muscle-strengthening physical activity guidelines (guidelines)* (*1*) during leisure time in nearly all demographic and regional subgroups 1998–2018 (*2*,*3*), differences by rurality and U.S. Census Bureau region (Northeast, Midwest, South, and West), persist (*4*). Before 2020, analyses of rural-urban differences were dichotomized into nonmetropolitan (rural) versus metropolitan (urban) areas; however, in 2020 a four-category rural-urban variable^†^ to classify rural-urban status was included in the National Health Interview Survey (NHIS) public-use dataset. NHIS 2020 data were used to conduct multivariate logistic regression analyses by rural-urban status and U.S. Census Bureau region of the prevalence of meeting the aerobic, muscle-strengthening, and combined aerobic and muscle-strengthening guidelines during leisure time among adults aged ≥18 years, controlling for demographic characteristics. Prevalence of meeting the aerobic, muscle-strengthening, and combined aerobic and muscle-strengthening guidelines was consistently the lowest in Nonmetropolitan counties (38.2%, 21.1%, and 16.1%, respectively) and highest in the West region (52.1%, 35.3%, and 28.5%, respectively). Regardless of rural-urban classification and region, no more than 28% of adults met combined aerobic and muscle-strengthening guidelines. Adults in the most rural category were significantly less likely to meet aerobic, muscle-strengthening, and combined guidelines than were adults in each of the three other categories (adjusted odds ratio [aOR] range = 0.68–0.89). In addition, adults in medium and small metropolitan counties were less likely to meet guidelines than were adults in the two most urban categories (aOR range = 0.85–0.89). Adults in the Northeast, Midwest, and South U.S. Census Bureau regions were less likely to meet guidelines than were adults in the West region (aOR range = 0.75–0.82). These analyses identify geographic disparities in leisure-time physical activity where focused population-level intervention efforts could help reduce or eliminate the consequent disparities in chronic conditions (e.g., cardiovascular diseases) and the resulting mortality (*5*,*6*).

NHIS is a nationally representative sample of noninstitutionalized U.S. adults that includes annual multistage cross-sectional household surveys conducted by CDC.^§^ NHIS 2020 public-use data were analyzed, because changes in the NHIS questionnaire precluded analysis of trend data or combining administration years. NHIS 2020 is also the first year that the NHIS public-use dataset included the four-category rural-urban county classification variable in public-use data. The 2020 sample of 31,568 adults included 21,153 (67%) participants interviewed for the 2020 annual administration and 10,415 (33%) from the 2019 sample who were reinterviewed for longitudinal analyses. Response rates for the 2020 sample were 48.9% (interviewed) and 29.6% (reinterviewed).^¶^ Among adults in the 2020 sample, information on the indicators of interest was missing for 1,161 (4%) respondents, resulting in a final analytic sample of 30,407.

Three dependent variables were analyzed. First, respondents were classified as either meeting or not meeting the aerobic guideline of ≥150 minutes per week based on self-reported frequency and duration of moderate and vigorous intensity leisure-time aerobic activity.** Second, respondents were classified as either meeting or not meeting the muscle-strengthening guideline of ≥2 days per week based on self-reported frequency of muscle-strengthening activities.^††^ Finally, respondents were classified as meeting the combined guideline if they met both the aerobic and muscle-strengthening guidelines.

Multivariable logistic regression analyses were conducted to model unadjusted and adjusted predicted population probabilities of dependent variables by rural-urban classification (nonmetropolitan [micropolitan and noncore], medium and small metropolitan, large fringe metropolitan, and large central metropolitan [referent]) and U.S. Census Bureau region (Northeast, Midwest, South, and West [referent]),^§§^ while controlling for biologic sex, age, race and ethnicity, education, and income-to-poverty threshold.^¶¶^ In addition, least-squares mean estimates were used to calculate the predicted population margin effects to compare within categories of the primary predictors (rurality and region). All analyses were performed using SAS (version 9.4; SAS Institute) with parameters adjusted for population weights, clusters, and stratification following NHIS analytic guidelines. These analyses were not subject to Institutional Review Board approval because deidentified public-use data were analyzed. This activity was reviewed by CDC and was conducted consistent with applicable federal law and CDC policy.***

Prevalence rates are 31.9%-72.3% higher in the most active counties by rural-urban classification and 20.3%-29.5% higher in the West than in the South U.S. Census Bureau region (Table 1). The lowest prevalence of meeting the aerobic, muscle-strengthening, and combined guidelines was observed among adults living in the most rural counties (nonmetropolitan; 38.2%, 21.1%, and 16.1%, respectively) and in the South U.S. Census Bureau region (43.3%, 29.0%, and 22.0%, respectively). Residents of medium and small metropolitan counties and nonmetropolitan counties were significantly less likely to meet aerobic, muscle-strengthening, and combined guidelines than were residents of large central metropolitan counties (aOR = 0.68–0.89). Compared with residents of the West U.S. Census Bureau region, those in all other U.S. Census Bureau regions were significantly less likely to meet aerobic, muscle-strengthening, and combined guidelines (aOR range = 0.75–0.82).

**TABLE 1:** Prevalence and main effect estimates of U.S. adults aged ≥18 years who met 2018 aerobic, muscle-strengthening, and combined physical activity guidelines during leisure time — National Health Interview Survey, United States, 2020

Characteristic	Met the 2018 physical activity guidelines								
	Aerobic			Muscle-strengthening			Both aerobic and muscle-strengthening		
	%*	OR	aOR^†^ (95% CI)	%*	OR	aOR^†^ (95% CI)	%*	OR	aOR^†^ (95% CI)
**Rural-urban classification** ^§^									
Nonmetropolitan	38.2	0.62	0.79 (0.71–0.89)^¶^	21.1	0.49	0.68 (0.60–0.77)^¶^	16.1	0.50	0.73 (0.63–0.83)^¶^
Medium and small metro	45.1	0.82	0.89 (0.81–0.98)^¶^	29.5	0.77	0.87 (0.78–0.97)^¶^	22.3	0.75	0.85 (0.77–0.94)^¶^
Large fringe metro	50.4	1.02	1.01 (0.93–1.11)	33.1	0.91	0.94 (0.86–1.03)	26.9	0.96	0.99 (0.90–1.10)
Large central metro	50.0	Ref	—	35.2	Ref	—	27.8	Ref	—
U.S. Census Bureau region**									
Northeast	47.9	0.85	0.80 (0.72–0.90)^¶^	30.9	0.82	0.81 (0.71–0.93)^¶^	24.4	0.81	0.77 (0.68–0.88)^¶^
Midwest	47.0	0.82	0.80 (0.72–0.89)^¶^	29.9	0.78	0.81 (0.73–0.89)^¶^	23.4	0.77	0.77 (0.68–0.86)^¶^
South	43.3	0.70	0.75 (0.69–0.82)^¶^	29.0	0.75	0.82 (0.74–0.91)^¶^	22.0	0.71	0.76 (0.69–0.85)^¶^
West	52.1	Ref	—	35.3	Ref	—	28.5	Ref	—

**Abbreviation:** aOR = adjusted odds ratio; MSA = metropolitan statistical area; OR = unadjusted odds ratio; Ref = referent group.

* Prevalence adjusted for population weights, clusters, and stratification following National Health Interview Survey analytic guidelines.

^†^ Adjusted for biological sex, age, race and ethnicity, education, and income-to-poverty threshold.

^§^ Nonmetropolitan = micropolitan counties (counties in micropolitan statistical areas) and noncore counties (counties that did not qualify as micropolitan); medium metro = counties in MSAs of populations of 250,000–999,999; small metro = counties in MSAs of populations less than 250,000; large fringe metro = counties in MSAs of 1 million or more population that did not qualify as large central metro counties; large central metro = counties in MSAs of 1 million or more population that 1) contain the entire population of the largest principal city of the MSA, or 2) have their entire population contained in the largest principal city of the MSA, or 3) contain at least 250,000 inhabitants of any principal city of the MSA. https://www.cdc.gov/nchs/data/series/sr_02/sr02_166.pdf

**^¶^** p≤0.01.

** https://www2.census.gov/geo/pdfs/maps-data/maps/reference/us_regdiv.pdf

In addition, least-squares mean estimates indicate that residents of nonmetropolitan counties were less likely to meet aerobic, muscle-strengthening, and combined guidelines than were residents of medium and small metropolitan counties (aOR range = 0.78–0.89) and large fringe metropolitan counties (aOR range = 0.72–0.78) (Table 2). Residents of medium and small metropolitan counties were less likely than were residents of large fringe metropolitan counties to meet aerobic (aOR = 0.88) and combined guidelines (aOR = 0.86). Residents in the Northeast, Midwest, and South regions did not differ from one another in likelihood of meeting guidelines (aOR range = 0.99–1.07).

**TABLE 2:** Comparison of U.S. adults aged ≥18 years who met 2018 aerobic, muscle-strengthening, and combined physical activity guidelines during leisure time, by rural-urban classifications and U.S. Census Bureau regions — National Health Interview Survey, United States, 2020

Comparison	Met the 2018 physical activity guidelines, aOR* (95% CI)		
	Aerobic	Muscle-strengthening	Both aerobic and muscle-strengthening
Rural-urban classification^†^			
Nonmetropolitan vs. medium/small metro	0.89 (0.80–0.99)^§^	0.78 (0.69–0.88)^¶^	0.85 (0.75–0.98)^§^
Nonmetropolitan vs. large fringe metro	0.78 (0.70–0.88)^¶^	0.72 (0.64–0.81)^¶^	0.73 (0.64–0.84)^¶^
Nonmetropolitan vs. large central metro	0.79 (0.71–0.89)^¶^	0.68 (0.60–0.77)^¶^	0.73 (0.63–0.83)^¶^
Medium/small metro vs. large fringe metro	0.88 (0.80–0.96)^¶^	0.93 (0.84–1.03)	0.86 (0.77–0.95)^§^
Medium/small metro vs. large central metro	0.89 (0.81–0.98)^§^	0.87 (0.78–0.97)^¶^	0.85 (0.77–0.94)^¶^
Large fringe metro vs. large central metro	1.01 (0.93–1.11)	0.94 (0.86–1.03)	0.99 (0.90–1.10)
U.S. Census Bureau region**			
Northeast vs. Midwest	1.00 (0.89–1.12)	1.00 (0.88–1.14)	1.01 (0.89–1.15)
Northeast vs. South	1.07 (0.96–1.18)	0.99 (0.87–1.13)	1.01 (0.90–1.14)
Northeast vs. West	0.80 (0.72–0.90)^¶^	0.81 (0.71–0.93)^¶^	0.77 (0.68–0.88)^¶^
Midwest vs. South	1.07 (0.97–1.18)	0.99 (0.90–1.09)	1.00 (0.90–1.11)
Midwest vs. West	0.80 (0.72–0.89)^¶^	0.81 (0.73–0.90)^¶^	0.77 (0.68–0.86)^¶^
South vs. West	0.75 (0.69–0.82)^¶^	0.82 (0.74–0.91)^¶^	0.76 (0.69–0.85)^¶^

**Abbreviations:** aOR = adjusted odds ratio; MSA = metropolitan statistical area.

* Adjusted for biological sex, age, race and ethnicity, education, and income-to-poverty threshold.

^†^ Nonmetropolitan = micropolitan counties (counties in micropolitan statistical areas) and noncore counties (counties that did not qualify as micropolitan); medium metro = counties in MSAs of populations of 250,000–999,999; small metro = counties in MSAs of populations less than 250,000; large fringe metro = counties in MSAs of 1 million or more population that did not qualify as large central metro counties; large central metro = counties in MSAs of 1 million or more population that 1) contain the entire population of the largest principal city of the MSA, or 2) have their entire population contained in the largest principal city of the MSA, or 3) contain at least 250,000 inhabitants of any principal city of the MSA. https://www.cdc.gov/nchs/data/series/sr_02/sr02_166.pdf

^§^ p≤0.01.

^¶^ p≤0.05.

** https://www2.census.gov/geo/pdfs/maps-data/maps/reference/us_regdiv.pdf

## Discussion

In 2020, the prevalence of meeting aerobic, muscle-strengthening, and combined physical activity guidelines in leisure time was lower among adults in nonmetropolitan versus metropolitan counties and higher in the West U.S. Census Bureau region than all other regions, suggesting persistent disparities in this important health behavior (*2*–*4*). In addition, because of the more detailed categorization within metropolitan (urban) counties, these analyses also identified differences in prevalence of meeting guidelines between more and less populated metropolitan counties. However, across all geographic and rural-urban categories, adherence to guidelines was low, with no more than 52% of adults meeting aerobic guidelines, 35% meeting muscle strengthening, and 28% meeting combined guidelines.

National efforts such as CDC’s Active People, Healthy Nation^†††^ and Healthy People 2030^§§§^ require ongoing, detailed surveillance to understand geographic disparities in meeting guidelines. Additional stratification by age, race and ethnicity, sex, income, and other characteristics (*7*) are important subsequent analyses needed to improve understanding of disparities and inform interventions to eliminate those disparities. Furthermore, physical activity prevalence data for narrower geographic areas (e.g., county and city) could provide evidence to guide local efforts to promote physical activity and ameliorate disparities. Ideally, these data would include the entire spectrum of physical activity intensities (i.e., sedentary, light, moderate, and vigorous) and purposes (i.e., leisure, occupational, transportation, and household).

Collective efforts to increase population-level physical activity in rural areas and small towns could benefit from using a conceptual framework to measure performance of the public health system as proposed by Illinois researchers in 2001 (*8*). This framework suggests that the successful implementation of services and achievement of population-level outcomes are a function of structural capacity of the public health system, which is constrained by the availability and use of human, informational, organizational, physical, and fiscal resources. Suggestions for increasing structural capacity for physical activity promotion in rural areas and small towns include enhancement of human and informational resources for rural physical activity programming. One approach to this is to develop practice-based evidence of novel partners (e.g., public librarians, barbers and hair stylists, and community health workers) who are successfully engaging in physical activity programming in rural areas and small towns, and then disseminate best practices tailored to these professionals in other areas of similar rurality and population size. A second approach includes providing professional development opportunities to established partners (e.g., health departments and Cooperative Extension) regarding current evidence-based practices for rural physical activity promotion. Such efforts to increase the number and variety of entities engaged in physical activity promotion could facilitate enhancement of organizational resources and advance the national, state, and local physical activity planning efforts that engage multisector coalitions (*9*). In addition, physical resources (i.e., the built environment) could be enhanced by translating evidence from research to inform community health improvement programming, abandoned mine land and brownfield remediation (i.e., removing or sealing points of contamination within a property so that it can be used without health concerns), and rural economic development to focus on physical activity–supportive built environment change.^¶¶¶^ Public, private, and philanthropic investments are necessary to support each of the other resources and build capacity in the system. Supporting local, state, and national research and practice networks, coalitions, and initiatives focused on population-level physical activity change in rural areas where physical activity prevalence is the lowest could help achieve the Active People, Healthy Nation goal of helping 27 million U.S. persons become more physically active by 2027.

The findings in this report are subject to at least three limitations. First, NHIS data collection occurred during the COVID-19 pandemic response, which has affected health behaviors such as physical activity (*10*). Second, self-reported physical activity is prone to recall bias and overestimation. Finally, lack of assessment of physical activity in other domains such as transportation, occupation, and household precluded the assessment of total physical activity.

This body of epidemiologic evidence is important for understanding rural-urban disparities in physical activity and tracking the attainment of national objectives; however, it is only the first step. A national paradigm shift is needed to build structural capacity through investments in human, informational, organizational, fiscal, and physical resources (*8*) and to implement policy, systems, and environment changes to impact population level physical activity across the United States, and especially outside of large metropolitan areas.

**Summary**

**What is already known about this topic?**

Physical activity is important in health promotion and disease prevention; rural-urban and regional disparities among adults in meeting the combined leisure time physical activity guidelines exist.

**What is added by this report?**

Analysis of 2020 National Health Interview Survey data found a low proportion of U.S. adults met leisure-time aerobic, muscle-strengthening, and combined physical activity guidelines. Residents in larger metropolitan areas and in the West U.S. Census Bureau region were more likely than were those in less populated urban and rural areas or other regions to meet these guidelines.

**What are the implications for public health practice?**

Rural residents might benefit from investments in structural capacity and policy, systems, and environment change to support leisure-time physical activity.

## Footnotes

* Adult aerobic physical activity guidelines include achieving ≥150 minutes of moderate-intensity physical activity per week, or ≥75 minutes of vigorous-intensity physical activity per week, or an equivalent combination of moderate-intensity physical activity and vigorous-intensity physical activity. Muscle-strengthening guidelines for adults include ≥2 days per week of activities of moderate or greater intensity that involve all major muscle groups.

† Nonmetropolitan = micropolitan counties (counties in micropolitan statistical areas [MSAs]) and noncore counties (counties that did not qualify as micropolitan); medium metro = counties in MSAs of populations of 250,000–999,999; small metro = counties in MSAs of populations less than 250,000; large fringe metro = counties in MSAs of 1 million or more population that did not qualify as large central metro counties; large central metro = counties in MSAs of 1 million or more population that 1) contain the entire population of the largest principal city of the MSA, or 2) have their entire population contained in the largest principal city of the MSA, or 3) contain at least 250,000 inhabitants of any principal city of the MSA. https://www.cdc.gov/nchs/data/series/sr_02/sr02_166.pdf

§
https://www.cdc.gov/nchs/nhis/index.htm

¶
https://ftp.cdc.gov/pub/Health_Statistics/NCHS/Dataset_Documentation/NHIS/2020/srvydesc-508.pdf

** Physical activity prompts: Frequency of moderate-intensity activity: “How often do you do moderate-intensity leisure-time physical activities?” Duration: “About how long do you do these moderate leisure-time physical activities each time?” Frequency of vigorous-intensity activity: “How often do you do vigorous-intensity leisure-time physical activities?” Duration: “About how long do you do these vigorous leisure-time physical activities each time?” Duration and frequency were multiplied to obtain weekly minutes, and minutes of vigorous-intensity activity were multiplied by 2 to equilibrate with moderate-intensity minutes.

†† Frequency of muscle-strengthening activity: “Including activities that you mentioned earlier, how often do you do leisure-time physical activities specifically designed to strengthen your muscles such as sit-ups, push-ups, or lifting weights?”

§§
https://www.cdc.gov/nchs/data/series/sr_02/sr02_166.pdf

¶¶ The RATCAT_A variable from the public-use NHIS Sample Adult file was used for analyses.

*** 45 C.F.R. part 46.102(l)(2), 21 C.F.R. part 56; 42 U.S.C. Sect, 241(d); 5 U.S.C. Sect. 552a; 44 U.S.C. Sect. 3501 et seq.

†††
https://www.cdc.gov/physicalactivity/activepeoplehealthynation/index.html

§§§
https://www.cdc.gov/nchs/healthy_people/hp2030/hp2030.htm

¶¶¶
https://www.cdc.gov/physicalactivity/community-strategies/activity-friendly-routes-to-everyday-destinations.html
